# Enhanced functional connectivity of the default mode network (DMN) in patients with spleen deficiency syndrome: A resting-state fMRI study

**DOI:** 10.1097/MD.0000000000014372

**Published:** 2019-02-01

**Authors:** Yan-zhe Ning, Feng-zhi Wu, Song Xue, Dong-qing Yin, Hong Zhu, Jia Liu, Hong-xiao Jia

**Affiliations:** a The National Clinical Research Center for Mental Disorders & Beijing Key Laboratory of Mental Disorders, Beijing Anding Hospital, Capital Medical University; b Advanced Innovation Center for Human Brain Protection, Capital Medical University; c Beijing University of Chinese Medicine; d State Key Laboratory of Cognitive Neuroscience and Learning, Beijing Normal University, Beijing, China.

**Keywords:** default model network, resting-state fMRI, spleen deficiency syndrome

## Abstract

Numerous studies had investigated the biological basis of spleen deficiency syndrome on gastrointestinal dysfunctions. However, little was known about neuropsychological mechanism of spleen deficiency syndrome. The default model network (DMN) plays an important role in cognitive processing. Our aim is to investigate the change of neuropsychological tests and DMN in patients with spleen deficiency syndrome.

Sixteen patients and 12 healthy subjects underwent functional magnetic resonance imaging examination, and 15 patients with spleen deficiency syndrome and 6 healthy subjects take part in the two neuropsychological tests.

Compared with healthy subjects, patients with spleen deficiency syndrome revealed significantly increased functional connectivity within DMN, and significantly higher in the scores of 2-FT (*P* = .002) and 3-FT (*P* = .014).

Our findings suggest that patients with spleen deficiency syndrome are associated with abnormal functional connectivity of DMN and part of neuropsychological tests, which provide new evidence in neuroimaging to support the notion of TCM that the spleen stores Yi and domains thoughts.

## 1 Introduction

Spleen deficiency syndrome, also named as “Pi-deficiency zheng” in Chinese, is one of the most common syndromes of traditional Chinese medicine (TCM) in the chronic disease and subhealthy populations,^[1]^ which can not only result in the gastrointestinal dysfunctions, such as edema and diarrhea, but also result in impairments of cognitive function, such as hypomnesia. According to TCM, it is mainly caused by excessive fatigue, over thinking or eating disorders, and frequently treated with fortifying the spleen.

In the last decades, studies on intrinsic mechanisms of spleen deficiency syndrome mainly focused on the pathogenesis from genomics, proteomics, epigenomics and metabonomics levels.^[2]^ One study explored pathogenesis of spleen deficiency syndrome patients from miRNA levels and identified 11 candidate serum miRNAs.^[3]^ Another study revealed differential expressed proteins between spleen deficiency syndrome and damp-heat syndrome from proteomics levels.^[4]^ However, there are few studies on investigating the biological basis of spleen deficiency syndrome from neuropsychology.

As we know, cognitive tests could be influenced by the patient's education as well as the tester's skills. Fortunately, as a non-invasive method, functional magnetic response imaging (fMRI) has been a useful technique to explore the neurobiological mechanisms of diseases, such as mild cognitive impairment,^[5]^ migraine,^[6]^ type 2 diabetes mellitus^[7]^ and so on. A recent study focused on cerebral activity changes in different TCM patterns of psychogenic erectile dysfunction patients by using fMRI.^[8]^ Default mode network (DMN) is a set of brain regions activated during resting state,^[9,10]^ which is believed to play an important role in cognition and memory.^[11]^ Several fMRI studies have revealed specific functional connectivity changes in the DMN of stroke,^[12]^ Alzheimer's disease,^[13]^ depression^[14]^ and so on. However, little is known about the changes of DMN and neuropsychology in patients with spleen deficiency syndrome.

In the present study, we conducted a combination of fMRI and neuropsychological tests to characterize the abnormality in patients with spleen deficiency syndrome from the aspect of neuropsychology. Sixteen patients with spleen deficiency syndrome from subhealthy populations and 12 age, gender-matched healthy subjects as controls were recruited. Independent component analysis (ICA) was applied to compute the functional connectivity values within the DMN. We hypothesized that patients with spleen deficiency syndrome would show abnormal neuropsychological tests and functional connectivity of DMN in comparison with healthy subjects.

## 2 Materials and methods

### 2.1 Participants

Sixteen right-handed subjects (2 males, aged 25.69 ± 3.75 years) were diagnosed as spleen deficiency syndrome according to the clinical guideline of new drugs for traditional Chinese medicine and met the criteria below: with no organic pathologic changes; aged from 18 to 45 years old; duration for more than 6 months; no color blindness and other vision problems. The exclusion criteria were as follows: any MRI contraindications; with history of neurologic disorders and psychoses; with history of drug or alcohol abuse. Another 12 healthy subjects (2 males, aged 28.00 ± 3.05 years) were recruited free from any symptoms of spleen deficiency syndrome; with no history of psychiatric or neurologic disorders. This study was approved by the Anding Hospital of Beijing Ethics Committee. All subjects signed informed consents before inclusion in this study.

### 2.2 Neuropsychological tests

We apply two evaluation tools to neuropsychological tests. Fifteen patients with spleen deficiency syndrome and six healthy subjects take part in the two neuropsychological tests.

To assess the short-term memory, we apply Clinical Memory Scale (CMS), which is designed by the Institute of Psychology, Chinese Academy Sciences.^[15]^ The scale contains directed memory, associative memory, free recall of images, meaningless image recognition and associative memory of portraits. The first two sections are auditory memory tests. The instructions and stimuli were recorded on tape and released by a tape recorder. The middle two sections are visual memory tests. Tester showed the stimuli to participants according to the prescribed time. The last section is the combination of auditory and visual memory. The subjects tell the characteristics of the pictures while being presented the stimuli of the pictures. The memory quotient (MQ) is used to measure the memory level, which is calculated according to the score of CMS.

To assess the attention, we applied Continuous Performance Test (CPT) via the computer.^[16]^ This computer program has two major task components: the immediate memory task (IMT) and the delayed memory task (DMT). In the IMT, a sequence of 2-to 4-digit numbers (e.g., 5921) appear on the computer monitor one-at-a-time for 500 ms each, with a 500-msec intertrial interval. In the DMT, subjects also match identical numbers, but the period of time separating the stimuli to be compared is longer, because a series of three distractor stimuli appear in between the two stimuli to be compared. The default presentation sequence is IMT/DMT/IMT/DMT, and a complete testing session using the default condition lasts exactly 21.5 minutes. There are three types of stimuli that can be generated during a testing session: target, catch, and filler stimuli. Target stimuli are numbers presented that are identical to the numbers immediately preceding them. Responses to these stimuli are recorded as correct detections. Catch stimuli are numbers presented that closely resemble the numbers immediately preceding them. Filler stimuli are numbers presented that are novel, differing from the numbers immediately preceding them. Four assessment indicators are selected to evaluate the attention, including false trials (FT), actual number of responses (ANR), reaction time mean (RTM) and standard deviation (SD). Numeric types include double-digit numbers, three-digit numbers and four-digit numbers.

### 2.3 MRI acquisition

We acquired images by using a 3.0 Tesla MRI scanner (Trio 3.0; Siemens, Erlangen, Germany) in the Imaging Center for Brain Research at Beijing Normal University. Before scanning, all participants were asked to take a rest for 30 minutes and were instructed to stay still, keep eyes closed, and not to fall asleep during scanning. Foam head holders were immobilized to minimize head movements during scanning.

Prior to the functional scanning, high-resolution structural information for anatomical localization was collected by applying MRI sequences. We collected resting-state fMRI data by using a single-shot, gradient-recalled echo-planar imaging sequence with the following parameters: repetition time = 2000 ms, echo time = 30 ms, flip angle = 90°, matrix = 64 × 64, field of view = 200 mm × 200 mm, slice thickness = 3.5 mm, gap = 1 mm, 33 axial sections, and 240 volumes.

### 2.4 Experimental paradigm

In the current research, we employed a 480-second resting scan first, and then 250-second high-resolution structural scan.

### 2.5 Data processing and analyzing

We conducted data pre-processing using software Data Processing Assistant for Resting-State fMRI (DPARSF, http://rfmri.org/ DPARSF) and the statistical parametric mapping software (SPM8, http://www.fil.ion.ucl.ac.uk/spm). After the first 10 volumes were discarded for signal equilibrium, a total of 230 volumes for each subject were corrected for slice timing. The following steps were spatial realignment for head motions, normalization into the Montreal Neurological Institute template, resampling into 3 × 3 × 3 mm^3^ voxels, smoothing with a Gaussian kernel of 4 mm full width at half-maximum, spurious variances (head motion, ventricular and white matter signal and the derivatives of each of these signals) reduction. We removed linear trends from the time courses and processed with a band pass filter of 0.01 to 0.10 Hz.

The Group Independent component analysis (ICA) of fMRI Toolbox (GIFT, http://mialab.mrn.org/software/gift/) was applied. After 20 times randlnit and bootstrap, and regression operations, we extracted DMN from thirty components. To compare the patient group with the healthy subject group, *P* values were set as .05 for group comparisons. The results were conducted within the DMN mask corrected by Monte Carlo Simulations, iterated 5000 times, and cluster size > 48 voxels (DPABI, http://rfmri.org/dpabi).^[17]^

## 3 Results

### 3.1 Demographic and clinical information

Demographic data of all subjects were displayed in Table 1. Gender, age and educational level were conducted by applying Chi-square analysis (gender) and non-parametric tests (age and education level). The two groups showed no significant difference in gender (*P* = 1.000), age (*P* = .106) and education level (*P* = .106). Clinical data were summarized in Table 2. The scores of MQ in patients with spleen deficiency syndrome showed no statistical difference in comparison with healthy subjects. The results of CPT showed that patients with spleen deficiency syndrome were significantly higher than healthy subjects in the scores of 2-FT (*P* = .002) and 3-FT (*P* = .014).

**Table 1:**
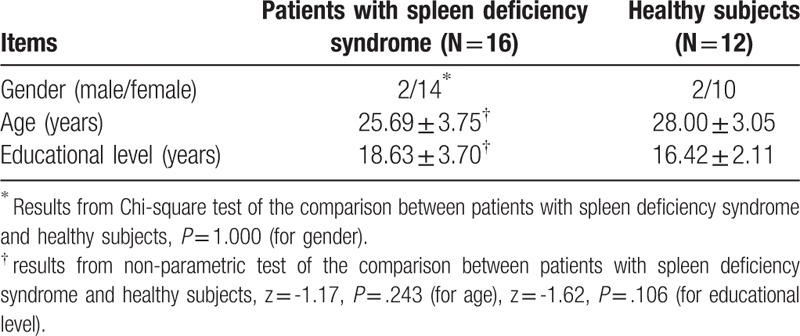
The demographic information of patients and healthy subjects.

**Table 2:**
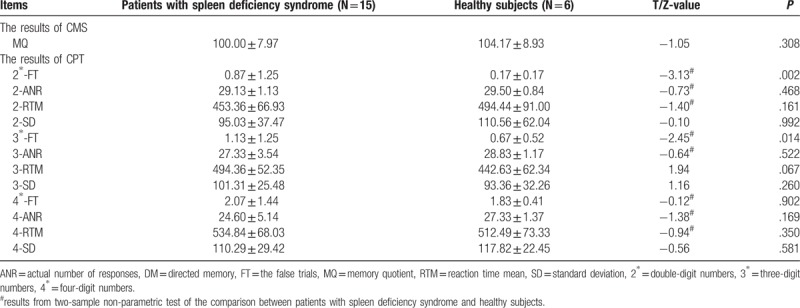
The results of neuropsychological tests between patients and healthy subjects.

### 3.2 FC analysis

**Figure 1:**
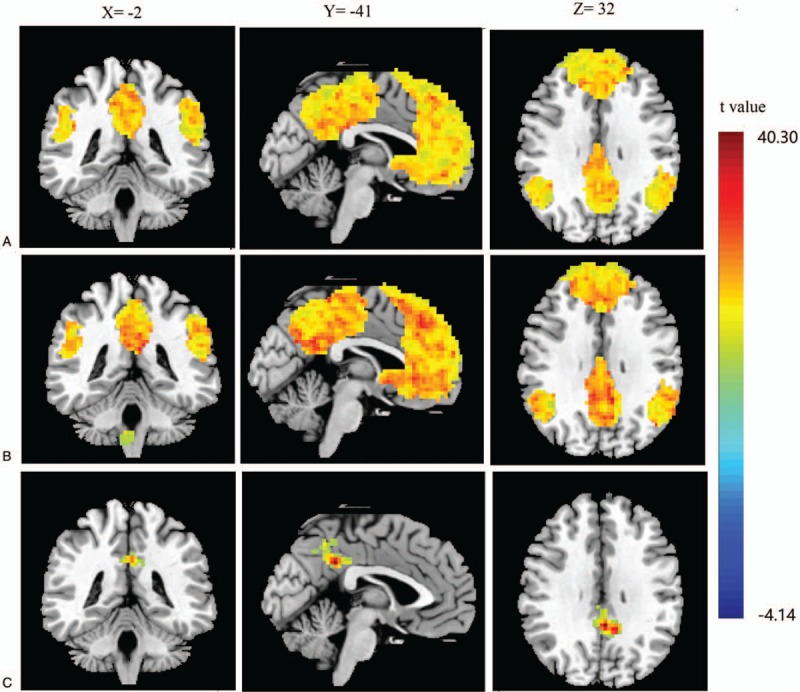
Altered functional connectivity of brain regions within DMN on patients with spleen deficiency syndrome and healthy subjects. (A) altered functional connectivity of brain regions within DMN on patients with spleen deficiency syndrome; (B) altered functional connectivity of brain regions within DMN on healthy subjects; (C) altered functional connectivity of brain regions within DMN between patients with spleen deficiency syndrome and healthy subjects. Results from one-sample *t* test and two-sample *t* test. (*P* < .05, corrected by Monte Carlo Simulations, iterated 5000 times, and cluster size > 48 voxels). DMN = Default mode network.

To detect the difference on functional connectivity of DMN between the patients with spleen deficiency syndrome and healthy subjects, two-sample T-test was applied between the two groups. During the resting state, compared with healthy subjects, the patients with spleen deficiency syndrome showed increased functional connectivity of DMN within the following brain regions such as the bilateral precuneus, bilateral cingulate gyri, central precuneus and central cingulate gyrus (shown in Fig. 1, *P* < .05, corrected by Monte Carlo Simulations, iterated 5000 times, and cluster size > 48 voxels). There was no decreased functional connectivity of brain region within DMN. Specific cluster locations, which represented altered functional connectivity of brain regions within DMN between patients with spleen deficiency syndrome and healthy subjects, were displayed in Table 3.

**Table 3:**
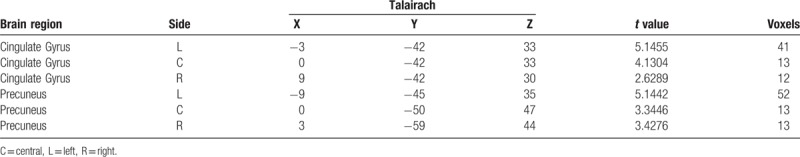
Results of altered functional connectivity of DMN between patients with spleen deficiency syndrome and healthy subjects.

## 4 Discussion

To the best of our knowledge, this is the first study to investigate the abnormal neuropsychology between patients with spleen deficiency syndrome and healthy subjects. In the current study, we found abnormal neuropsychological tests and increased functional connectivity within the DMN.

As we know, the clinical manifestations of spleen deficiency syndrome included poverty of thought, hypoprosexia and memory deficits, especially in short memory. Thus, we applied the CMS and CPT to assess the memory and attention. The result of MQ in patients with spleen deficiency syndrome did not show significant decrease compared with healthy subjects, which may be related with the small sample size of healthy subjects. However, the mean value of MQ in patients with spleen deficiency syndrome was lower than healthy subjects, which may partly indicate the decreased short memory in patients with spleen deficiency syndrome. Furthermore, there were significantly higher scores of 2-FT and 3-FT in patients with spleen deficiency syndrome compared with healthy subjects, which revealed that patients with spleen deficiency syndrome showed decreased attention.

According to the Yellow Emperor's Inner Classic (Huangdi Nei Jing), spleen stores Yi and domains thoughts. Yi is considered to include the memory and thought. Yi could not only form short-term memory, process information and develop the long-term memory, but also extract information from the long-term memory.^[18,19]^ In hence, once Yi is harmed, it will cause poverty of thought and decreased memory. Furthermore, according to TCM theory, the function of spleen mainly includes digestive function. As we know, there are intricate links between the gastrointestinal tract and the central nervous system via biochemical signals, which is referred as the “brain-gut axis” .^[20]^ The two system remain constantly exchange streams of chemical and electrical messages, and affect each other. Recent studies had demonstrated that patients with irritable bowel syndrome had abnormalities in brain structure and functional connectivity.^[21–23]^ Recently, it has become clear that the bidirectional communication pathway between gut bacteria and brain. The role of the gut microbiota in brain health was appreciated.^[24]^ Moreover, modulation of the gut microbiota can affect the severity of the central pathology or behavioral deficits observed in a variety of brain disorders, such as depression, autism, stroke, Parkinson disease, and Alzheimer's disease.^[25–28]^ Thus, it makes sense that there are the abnormalities in neuropsychology of patients with spleen deficiency syndrome according to the theory of brain-gut axis and microbiota–gut–brain axis.

Moreover, we found the abnormal functional connectivity within DMN between patients with spleen deficiency syndrome and healthy subjects. As we know, DMN is associated with the processing and integration of emotional, cognitive, physical information, and mind wandering,^[29]^ which covers the posterior cingulate, precuneus, lateral parietal area, mesial prefrontal cortex, as well as hippocampal formation.^[9,30]^ Functionally, the DMN is considered to be associated with episodic memory.^[11,31]^ Alzheimer's disease manifests the most common early symptom as short-term memory impairment.^[32]^ With the increasing studies on Alzheimer's disease from neuroimaging, DMN is drawn attention as a potential noninvasive biomarker for Alzheimer's disease.^[33,34]^ Accordingly, DMN may play a potential biomarker for patients with spleen deficiency syndrome from neuropsychology.

In our current study, the enhanced functional connectivity in DMN was found in patients with spleen deficiency syndrome, which was in line with related studies. The altered functional connectivity in DMN increased or decreased for different diseases. For stroke patients, decreased functional connectivity was found within DMN.^[12]^ Patients with attention deficit hyperactivity disorder also showed decreased functional connectivity within DMN.^[35]^ Moreover, one recent study revealed that functional connectivity in DMN increased from mild amnestic mild cognitive impairment (aMCI) to moderate aMCI.^[36]^ Another study on patients with paranoid schizophrenia also showed significant enhanced functional connectivity within DMN in comparison with healthy subjects.^[37]^ For late-life depression patients, increased functional connectivity of DMN was also found.^[38]^ Similarly, our results can be interpreted as the specific abnormality in brain function for patients with spleen deficiency syndrome. Our results may partly explain the impairments of cognitive function in patients with spleen deficiency syndrome compared with healthy subjects and are consistent with our predictions.

There were also some possible limitations to the current study. First, as a preliminary study, our sample size was relatively small (n = 16 patient group), which might affect the statistical power of results and result in no significant correlation between fMRI results and neuropsychological tests. Second, we recruited patients with spleen deficiency syndrome from subhealthy populations. It would be better to study the specific disease with spleen deficiency syndrome in the future, such as irritable bowel syndrome. Third, it would be better to use Chinese brain atlas during data preprocessing in our current study, due to morphological differences in Chinese and Caucasian Populations.

## 5 Conclusion

To our knowledge, the current study is the first to probe into the neuropsychological mechanism of spleen deficiency syndrome on the basis of resting-state brain networks by applying fMRI. Our results suggest that patients with spleen deficiency syndrome are associated with abnormal functional connectivity of DMN, and provide new evidence in neuroimaging to support the notion of TCM that the spleen stores Yi and domains thoughts.

## Author contributions

YZN, FZW, JL and HXJ conceived and designed the study. YZN and FZW analyzed the data. FZW, DQY, HZ and SX performed the experiment. YZN, FZW, JL and HXJ drafted the manuscript and gave final approval of the manuscript.

**Conceptualization:** hongxiao Jia, Yan-zhe Ning, Jia Liu.

**Data curation:** Feng-zhi Wu, Song Xue, Dong-qing Yin, Hong Zhu.

**Formal analysis:** Yan-zhe Ning, Dong-qing Yin.

**Funding acquisition:** hongxiao Jia.

**Validation:** Hong Zhu, Jia Liu.

**Writing – original draft:** Yan-zhe Ning.

**Writing – review & editing:** hongxiao Jia, Feng-zhi Wu, Jia Liu.
